# Parallel DNA pyrosequencing unveils new zebrafish microRNAs

**DOI:** 10.1186/1471-2164-10-195

**Published:** 2009-04-27

**Authors:** Ana R Soares, Patrícia M Pereira, Bruno Santos, Conceição Egas, Ana C Gomes, Joel Arrais, José L Oliveira, Gabriela R Moura, Manuel AS Santos

**Affiliations:** 1 RNA Biology Laboratory, Department of Biology & CESAM, University of Aveiro, 3810-193 Aveiro, Portugal; 2 Centre for Neuroscience and Cell Biology, University of Coimbra, 3004-517 Coimbra, Portugal; 3 Biocant Research Centre, 3060-197 Cantanhede, Portugal; 4 IEETA, University of Aveiro, 3810-193 Aveiro, Portugal

## Abstract

**Background:**

MicroRNAs (miRNAs) are a new class of small RNAs of approximately 22 nucleotides in length that control eukaryotic gene expression by fine tuning mRNA translation. They regulate a wide variety of biological processes, namely developmental timing, cell differentiation, cell proliferation, immune response and infection. For this reason, their identification is essential to understand eukaryotic biology. Their small size, low abundance and high instability complicated early identification, however cloning/Sanger sequencing and new generation genome sequencing approaches overcame most technical hurdles and are being used for rapid miRNA identification in many eukaryotes.

**Results:**

We have applied 454 DNA pyrosequencing technology to miRNA discovery in zebrafish (*Danio rerio*). For this, a series of cDNA libraries were prepared from miRNAs isolated at different embryonic time points and from fully developed organs. Each cDNA library was tagged with specific sequences and was sequenced using the Roche FLX genome sequencer. This approach retrieved 90% of the 192 miRNAs previously identified by cloning/Sanger sequencing and bioinformatics. Twenty five novel miRNAs were predicted, 107 miRNA star sequences and also 41 candidate miRNA targets were identified. A miRNA expression profile built on the basis of pyrosequencing read numbers showed high expression of most miRNAs throughout zebrafish development and identified tissue specific miRNAs.

**Conclusion:**

This study increases the number of zebrafish miRNAs from 192 to 217 and demonstrates that a single DNA mini-chip pyrosequencing run is effective in miRNA identification in zebrafish. This methodology also produced sufficient information to elucidate miRNA expression patterns during development and in differentiated organs. Moreover, some zebrafish miRNA star sequences were more abundant than their corresponding miRNAs, suggesting a functional role for the former in gene expression control in this vertebrate model organism.

## Background

MicroRNAs (miRNAs) are small RNAs that regulate eukaryotic gene expression at the post-transcriptional level [1]. They are transcribed as long precursor RNA molecules (pri-miRNAs) and are successively processed by two key RNAses, namely Drosha and Dicer, into their mature forms of ~22 nucleotides [2]. These small RNAs regulate gene expression by binding to target sites in the 3' untranslated region of mRNAs (3'UTR) [3,4]. Recognition of the 3'UTR by miRNAs is mediated through complementary hybridization between nucleotides 2–8, numbered from the 5' end (seed sequences) of the small RNAs, and complementary sequences present in the 3'UTRs of mRNAs [3,5,6]. Perfect or nearly perfect complementarities between miRNAs and their 3'UTRs induce mRNA cleavage by the RNA-induced silencing complex (RISC), whereas imperfect base matching induces translational silencing through various molecular mechanisms [7], namely inhibition of translation initiation and activation of mRNA storage in P-bodies and/or stress granules [1]. Interestingly, miRNAs also direct rapid deadenylation of target mRNAs, leading to decapping and rapid mRNA decay by the combined activities of the exosome (3' to 5' degradation) and the exoribonuclease Xrn1 (5' to 3'degradation) [7,8].

Since the discovery of the first miRNA in 1993 in *C. elegans *[9], thousands of mature miRNAs have been uncovered in several species, suggesting that they appeared early in eukaryotic evolution and play fundamental roles in gene expression control. Indeed, miRNAs have been identified using a combination of bioinformatics, cloning and Sanger sequencing, and lately through new generation sequencing methods, namely the Roche 454 Pyrosequencer, the Solexa/Illumina Genome Analyzer and the Applied Biosystems SOLiD™ Sequencer, in a wide range of eukaryotes, namely plants [10-13], mammals [14-16], birds [17,18], fish [19-21], amphibians [22], worms [23], flies [24], in the unicellular green algae *Chlamydomonas reinhardtii *[25] and in viruses [26]. These small RNAs were originally thought to regulate developmental processes only [27-29], but recent studies show that they regulate a variety of other pivotal biological processes, namely differentiation [30], immune response [31], infection [32,33] and cancer [34,35]. The exact mechanism by which they regulate such a variety of molecular processes is not yet fully understood, however 2–3% of the human genes encode miRNAs and approximately 30% of human mRNAs contain miRNA binding sites in their 3'UTRs, suggesting major roles for these small RNAs in eukaryotic gene regulation [36].

The quantification of miRNA expression has been technically challenging and rather expensive due to their small size, low abundance, low stability and contamination with other cellular RNAs and mRNA fragments. Recently, the above mentioned parallel DNA sequencing methodologies have been successfully applied to both miRNA identification and quantification [11,14,16,37]. The enormous sequencing power of these technologies has overcome most of the technical hurdles associated to miRNA identification and increased dramatically the number of miRNAs deposited in public databases [11]. These new methodologies are also promoting large scale initiatives to identify most eukaryotic miRNAs, understand their evolution and identify target genes and gene networks regulated by them.

In zebrafish (ZF), 337 miRNA genes encode 192 different mature miRNAs [38]. However, deep DNA sequencing has not yet been applied to this model organism and one is not sure whether those miRNAs represent the full ZF miRNA population [19,20]. As in other eukaryotes, recent ZF studies highlighted critical miRNA roles in gene expression control since defective miRNA processing arrested development [39,40]. Also, a specific subset of miRNAs is required for brain morphogenesis in ZF embryos, but not for cell fate determination or axis formation [41]. In other words, miRNAs play an important role in ZF organogenesis and their expression at specific time points is relevant to organ formation and differentiation. Since identification of the complete set of miRNAs is fundamental to fully understand these and other fundamental biological processes, we have used high throughput 454 DNA pyrosequencing technologies to fully characterize the ZF miRNA population. This study increased the total number of ZF miRNAs from 192 to 217 and identified several star sequences (miRNA*, complementary to miRNA sequences). In addition, miRNAs predicted by homology were retrieved and novel miRNA genes encoding known miRNAs were identified.

## Results

### 454 DNA sequencing of zebrafish miRNAs

**Figure 1:**
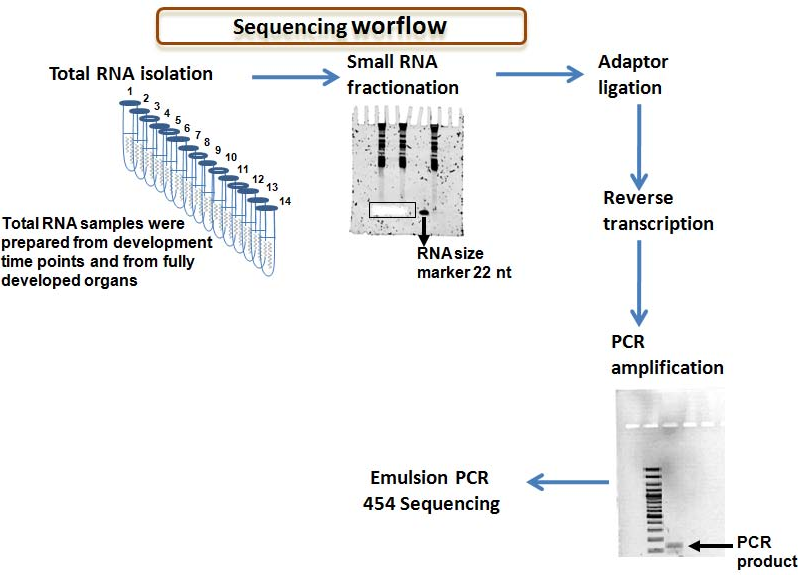
**Outline of the experimental protocol used for preparation of small RNA libraries**. RNA was isolated from ZF developmental samples and from adult tissues using TRIzol^® ^and fractionated on 12.5% denaturing PAGE. Small RNAs were purified from those gels and then ligated to a 3' adapter (AMP-5'p-5'p/CTGTAGGCACCATCAATdi-deoxyC- 3') and to a 5' linker (see Methods). cDNA was prepared and amplified using 20 PCR cycles. PCR products were subjected to clonal amplification by emulsion PCR and then pyrosequenced using a 454 genome sequencer.

In order to increase coverage of ZF miRNAs by 454 pyrosequencing, small RNA libraries were prepared from ZF samples collected at various developmental time points, i.e., 24 hours post-fertilization (hpf), 72 hpf, 96 hpf, 5 days post-fertilization (dpf), 45 dpf, and from young adult fish, adult brain, eyes, gills, muscle, heart, skin, fins and gut/liver (Figure 1). For this, total RNA from each sample was isolated with TRIzol^® ^and fractionated by PAGE. Small RNAs ranging from 15 to 30 nt in size were isolated from gels and subjected to two successive ligations, i.e., a first ligation with a 3' adapter was followed by a second ligation with a 5' adapter (see Methods). First strand cDNA synthesis was then carried out and PCR-amplified using adapter specific primers. Specific tags for each cDNA library corresponding to different developmental stages and tissues were incorporated into the PCR primers. PCR products of ~100 nt in length were then purified from a 10% denaturing PAGE containing 7 M urea. The 14 PCR products, corresponding to different developmental stages and to different mature tissues were sequenced using massively parallel DNA pyrosequencing. Raw data filtration was performed using specialist software incorporated into the FLX Genome Sequencer (Roche) [42]. The above mentioned sequencing tags and the sequencing adapters were identified computationally. Reads with recognizable adapters were retrieved, adapter sequences were then removed, and those reads with size ≥ 15 nt were analysed using miRDeep software [43]. The latter scores the compatibility of the position and frequency of the RNA sequence with the secondary structure of the miRNA precursor and identifies new, conserved and non-conserved miRNAs with high confidence. miRDeep also estimates false positives by random permutation of the signature and structure-pairings in the input dataset to test the hypothesis that the structure (hairpin) of true miRNAs is recognized by Dicer and causes the signature. In our study, the prediction of false positives was below 8%. Since miRDeep is a highly stringent algorithm some miRNAs are likely to escape detection. To minimize this problem the false negative rate was also calculated. For this, our sequencing data set was subjected to a megaBlast search using known mature miRNAs present in miRBase 12.0. Perfect alignments were considered as true positives and the retrieved miRNA list was then compared with the list of sequences predicted by miRDeep. False negatives were considered when miRNAs were present in the blast analysis and missed in the miRDeep prediction. This estimated ~19% of false negatives in the miRDeep prediction list. For this reason, the search for novel and known miRNAs in our ZF samples was complemented by a megaBlast alignment between our dataset and mature sequences deposited in miRBase 12.0 and also the novel miRNA transcripts predicted by Ensembl and by Thatcher *et al *[44] (see Additional File 1).

**Figure 2:**
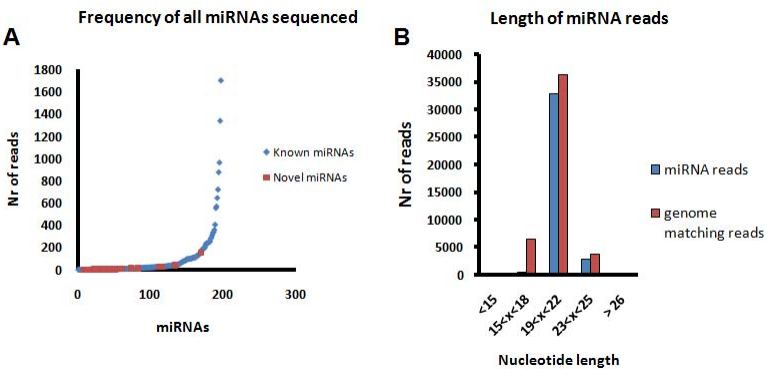
**A) Frequency of miRNAs sequenced**. The number of reads of each miRNA is shown. The contribution of each miRNA to the total number of reads was variable and a small number of miRNAs generated approximately half of the total number of reads. **B)** Size distribution of sequenced miRNAs. The length of reads matching the ZF genome ranged from 15 to 25 nucleotides. Most miRNA reads had between 19–22 nt in length, thus confirming the high quality of the small RNA libraries used in the pyrosequencing run.

A total of 67,044 high quality reads were obtained from cDNA libraries using pyrosequencing mini-chips (max nr reads = 100,000). From these, 63,637 had a recognizable TAG, 61,672 (size > 15 nt) and were retrieved after primer trimming, 46,904 matched the ZF genome using megaBlast and 36,989 corresponded to miRNA reads (Table 1). The majority of miRNA reads (~98%) matched known or predicted ZF miRNAs and less than 2% corresponded to putative novel miRNAs. This approach identified a total of 198 miRNAs: 173 of the known 192 mature miRNAs which are annotated in miRBase 12.0 plus 25 novel miRNAs. The identified miRNAs covered 90% of the known ZF miRNAs (Figure 2A; Table 2; see Additional File 2), previously identified by cloning and Sanger sequencing or predicted by bioinformatics (Table 3). The number of microRNA reads predicted by bioinformatics algorithms was rather low (average of 37) suggesting that the inability to detect and identify them by cloning and Sanger sequencing may be related to their low abundance.

**Table 1:** Distribution of microRNA reads in developmental and tissue samples.

	**Development stages**	**Mature organs**	**Total**
**Nr reads (perfect match to the genome)**	**20514**	**26390**	**46904**

**Nr non-miRNA reads**	2610	7305	9915

**Nr miRNA reads**	**17904**	**19085**	**36989**

**Nr known miRNA reads**	17682	18984	36666

**Nr putative new miRNA reads**	**222**	**101**	**323**

Approximately 90% of the pyrosequencing reads matching the ZF genome corresponded to mature miRNA reads, 2% were novel miRNAs. The distribution of pyrosequencing reads was similar in developmental (87%) and adult tissue (78%) samples.

**Table 2:** Total number of miRNAs and miRNA genes identified in this study.

**Number of known miRNAs detected**	**173**
Number of new miRNAs identified in this study	25
**Total number of miRNAs identified in this study**	**198**
Number of known predicted miRNA genes detected	265
Number of novel genes identified in this study	55
**Total of genes indentified in this study**	**320**

DNA pyrosequencing identified 198 miRNAs. 173 were known from cloning and Sanger sequencing and 25 were novel miRNAs. These mature miRNAs are encoded by 320 ZF genes.

**Table 3:** miRNAs predicted by homology and experimentally validated.

**miRNA**	miRNA* present	**Read number**
**Dre-let-7j**	No	9
**Dre-miR-103**	No	45
**Dre-miR-107**	Yes	32
**Dre-miR-135b**	No	5
**Dre-miR-152**	No	46
**Dre-miR-365**	Yes	76
**Dre-miR-429**	No	35
**Dre-miR-455**	Yes	41

Some of the known miRNAs identified in this study were predicted previously by comparative genomics using homology and similarity searches.

**Figure 3:**
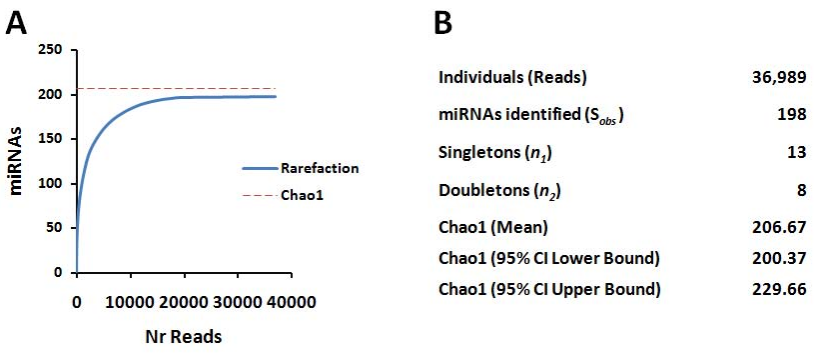
**Statistical analysis of miRNA population**. **A) **A rarefaction curve of the total number of reads generated by pyrosequening versus the total number of miRNA species identified is shown. The steep curve levels off towards an asymptote, which indicates the point where additional sampling will not yield new miRNAs. The stable value of 198 miRNAs validated our sampling methodology. **B) **Homogeneity of the ZF miRNA population was assessed using population statistics and by determining the Chao1 diversity estimator. The Chao1 reached a mean stable value of 207, with lower and upper limits of 200.37 to 229.66, respectively, for a level of confidence of 95%. The data indicates that the 198 miRNAs identified by pyrosequencing (173 known plus 25 new) plus 19 missed miRNAs (identified by Sanger sequencing), (total = 217 miRNAs) are near the upper limit of expected ZF miRNAs present in the pyrosequencing data set.

Reads matching the ZF genome (90%) were between 19 to 22 nt in length which corresponded to the mean length of mature miRNAs (Figure 2B). This high percentage of true miRNAs showed that inefficient amplification, sequencing or trimming errors did not affect our experiment [45]. Population statistics [46,47] was then applied to calculate the population of miRNAs expected in our dataset. Representativeness assessed through a rarefaction analysis [48] of the ZF miRNA population estimated a population size of 198 different miRNAs (Figure 3A). The homogeneity of the ZF miRNA population was evaluated by the Chao1 diversity estimator [49], which indicated the maximum number of miRNAs expected for the pyrosequencing dataset. The Chao1 reached a stable mean value of 207, with lower and upper bounds of 200.37 and 229.66, respectively, for 95% confidence interval. This was in good agreement with the 217 ZF miRNAs identified: 198 miRNAs identified by the 454 pyrosequencing approach (173 known and 25 new) plus 19 miRNAs previously identified by cloning and Sanger sequencing (not identified in our experiment). In other words, the total number of identified miRNAs is near the upper limit of expected ZF miRNAs in the samples studied (Figure 3B). However, one cannot discard the hypothesis that some novel miRNAs may still be uncovered in tissues that were not analyzed in this study.

Since 14,768 pyrosequencing reads did not match the ZF genome, we repeated the alignment using the mapping algorithm SHRiMP, which allows for introduction of insertions/deletions in the alignment. In this case, 87% (13,046 reads) of those reads produced matches in the ZF genome, however novel miRNAs were not identified. This analysis revealed some sequence variation in known miRNAs which may be related to sequencing errors or eventually to post-transcriptional miRNA editing [50]. For example, 14 reads of the dre-miR-124 family had a (C->A) substitution at position 20, but the low number of reads of each sequence did not permit unequivocal differentiation between miRNA editing and sequencing errors. This should be investigated further in a new study.

### miRNA expression patterns in zebrafish

The 24 hpf sample, corresponding to the developmental sample series, yielded low number of sequencing reads corresponding to 13 different miRNAs. The number of reads increased dramatically at late stages of development and 72 hpf sample produced the highest number of reads and the highest miRNA diversity (149 unique miRNAs) (see Additional File 3). At 96 hpf lower number of reads and lower miRNA diversity was observed. As ZF reached the adult stage, the number of reads and the number of different miRNAs raised again, which was consistent with previous studies [40,51]. This suggested that miRNAs play an important role in differentiation and maintenance of tissue identity, rather than in tissue fate establishment [40]. In adult fish, the brain sample produced the highest number of reads and the highest miRNA diversity (160 unique miRNAs). The gut/liver showed lower miRNA diversity (55 unique miRNAs) and the skin produced the lowest number of reads corresponding to 58 different miRNAs (see Additional File 3).

**Figure 4:**
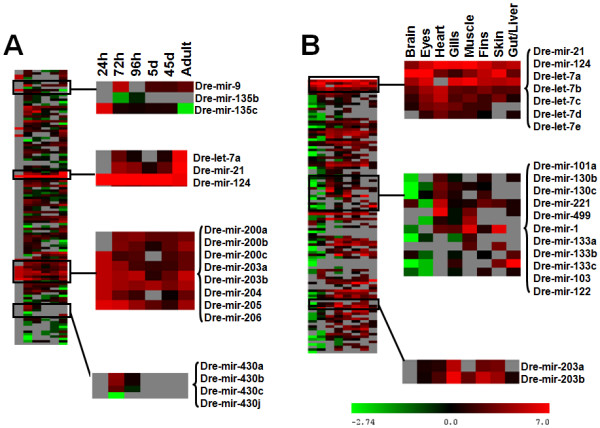
**Zebrafish miRNA expression profiles**. Development **(A)** and mature tissue **(B)** expression profiles generated by MeV 4.0 software using normalized reads number of each miRNA are shown. Some miRNAs were mainly expressed during development, namely dre-miR-430 family, dre-miR-135c and dre-miR-9. The former was absent in the adult fish while dre-miR-135c and dre-miR-9 had decreased expression in mature organs with exception of the brain. Conversely, dre-miR-124, dre-let-7a and dre-mir-21 were ubiquitously expressed. Dre-miR-499 was heart specific, dre-miR-1 and dre-miR-133a were detected in both muscle and heart and dre-miR-103 and dre-miR-122 were gut/liver specific.

Although the number of reads can be used to estimate miRNA abundance (expression profile) the variation in the total number of reads between samples would lead to erroneous interpretation of miRNA expression patterns by direct comparison of absolute read numbers. To overcome this limitation, the number of reads per sample was normalized, as described by Chen and colleagues [19], and expression of some miRNAs was validated by quantitative Real-Time PCR (qRT-PCR). After this, a global miRNA expression profile was generated for ZF (Figure 4). A large set of miRNAs were expressed in more than one tissue while some were mainly expressed during development (Figure 4A). Others were tissue specific or showed strong expression in specific tissues (Figure 4B). For example, the miR-430 family, dre-miR-135c and dre-miR-9 were mainly expressed during development, but the miR-430 family was absent in adult fish, while dre-miR-135c and dre-miR-9 showed decreased expression in mature organs with exception of the brain.

**Figure 5:**
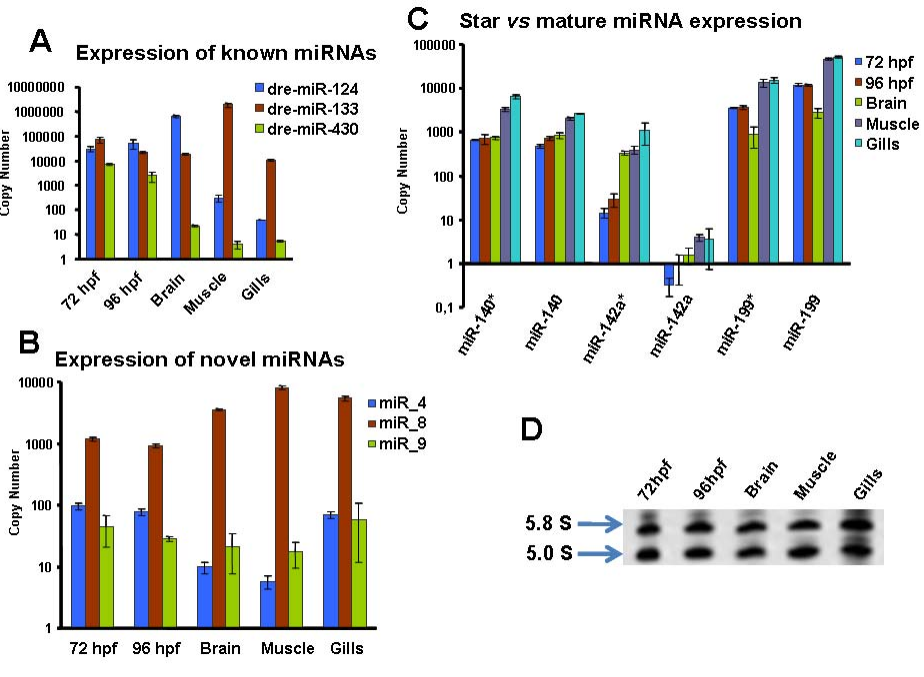
**qRT-PCR analysis of miRNA expression**. **A)** Expression of known miRNAs. Dre-miR-124 expression was higher in developmental and brain samples. Dre-miR-133 expression was higher in muscle and dre-miR-430 showed higher expression in developmental samples. **B)** Expression of novel miRNAs. miR_8 expression was higher in differentiated organs, miR_4 and miR_9 displayed similar expression levels throughout development and in differentiated organs. **C)** Star vs mature miRNA expression. The expression of dre-miR-140* and dre-miR-199* was similar to that of their respective mature miRNAs. Dre-miR-142* showed significantly higher expression than its mature miRNA, which was not detected during development. **D)** 5.8 S and 5.0S RNA samples. Denaturing 12% acrylamide gel showing the relative concentration of 5.0S and 5.8S RNA in the samples used in the qRT-PCR.

Of the 173 known miRNAs, which were also sequenced in this study, some were highly represented in all samples. For example, dre-miR-124 was the most abundant miRNA during both development and in adult ZF. Its expression was slightly higher during late stages of development and highly increased in the central nervous system (both brain and eyes), as confirmed by qRT-PCR (Figure 5). This miRNA alone accounted for ~48% of the total number of sequencing reads. At 24 hpf, when a significant part of the brain development was completed, dre-miR-124 represented 42% of the miRNA pool. These values increased to 80% at 5 dpf and also in mature tissues where it represented 80% of brain and 54% of eye miRNAs [19]. Members of the let-7 family and dre-miR-21 showed high levels of expression in the majority of tissues, e.g., dre-miR-21 yielded 21% of muscle miRNAs. Finally, some miRNAs were enriched in development and in a particular adult tissue; dre-miR-203a and dre-miR-203b were expressed early in development and maintained high level of expression in gills and skin. The heart showed accumulation of dre-miR-101a, dre-miR-130b, dre-miR-130c, dre-miR-221 and dre-miR-499, while dre-miR-1 and dre-miR-133a expression was detected in both muscle and heart. The expression of miR-133a was confirmed by qRT-PCR and its relative concentration was higher in muscle than in other tissues (Figure 5). Dre-miR-133b and dre-miR-133c were mainly found in muscle and were not detected in the heart, while dre-miR-103 and dre-miR-122 were specific of gut/liver and dre-miR-150 and dre-miR-738 were enriched in gills and skin.

### Novel zebrafish miRNAs are mostly non-conserved

A large set of both conserved and non-conserved miRNAs were previously identified in ZF by cloning/Sanger sequencing [19,20], using miRNA cDNA libraries prepared mainly from brain and from few developmental stages, and also using bioinformatics [44]. Our approach of isolating and preparing separated miRNA cDNA libraries from 24 hpf, 72 hpf, 96 hpf, 5 dpf, 45 dpf, total adult and from brain, eyes, heart, gills, muscle, fins, skin and gut/liver resulted in identification of 25 new miRNAs (see Additional File 4), using the miRDeep software package in combination with our data pipeline analysis (see Additional File 1). The miRDeep algorithm performed stringent searches based on the miRNA biogenesis model [43] and produced information on miRNA conservation, thermodynamic stability, ability to form a hairpin with the shape and sequence of the pre-miRNA molecule, number of sequences that matched mature miRNA sequences and number of sequences that matched star sequences. This algorithm alone was able to detect 153 known and 23 novel miRNAs. Our data pipeline (see Additional File 1) identified 20 known miRNAs missed by miRDeep in our samples, resulting in a total of 173 known miRNAs identified. The extension of this analysis to sequences predicted by Ensembl and by Thatcher *et al *[44] permitted detection of two additional novel miRNAs, raising the prediction number to 25.

The 25 novel miRNAs belong to 16 novel candidate miRNA families (16 novel miRNAs in total) and to 8 conserved miRNA families, according to our conservation criteria based in previous studies [20,52] (> 90% identity for the mature miRNA and > 60% identity for the precursor). The conservation of the novel miRNAs was confirmed by blast analysis against miRBase 12.0 and Ensembl database. The novel conserved miRNAs identified also showed 100% identity in the seed sequence between nucleotides 2 and 8. This criterion is largely used when assessing miRNA conservation [52,53], simultaneously with the mature and precursor identity. Interestingly, the conserved novel miRNAs retrieved by miRDeep, namely miR_4 (miR-429 family), miR_5 (miR-429 family), miR_6 (miR-1788 family), miR_11 (miR-196 family), miR_15 (miR-196 family), miR_16 (miR-103 family) and miR_21 (miR-222 family) were also predicted as novel ZF miRNAs by Ensembl algorithms (Table 4). miR_17 (miR-455 family), and miR_25 (miR-126 family), although not retrieved by miRDeep, were considered putative novel miRNAs since they were also predicted by Ensembl algorithms after applying our complementary analysis. Most of these novel miRNAs were detected throughout development and in adult tissues indicating that they may be involved in differentiation or maintenance of tissue identity [40]. Nine of the novel miRNAs started with uridine (U), which is characteristic of the first nucleotide position of mature miRNAs (see Additional File 4).

**Table 4:** Novel zebrafish miRNAs. miRNAs identified in this study, their level of conservation and corresponding miRNA families.

**miRNA ID**	**miRNA sequence**	**Conservation**	**miRNA family**	**miRDeep prediction**	**Ensembl prediction**
miR_1	AACAGTAAGAGTTTATGTGCTG	Non-conserved	-	√	No

miR _2	CGGTGCAGGACTCCGCGGCTC	Non-conserved	-	√	No

miR _3	AAGTGGCCTCTAAAAGTCTA	Non-conserved	-	√	No

miR _4	TAATACTGCCTGGTAATGCCAT	Conserved	miR-429	√	√

miR _5	ATCTCAGGTTCGTCAGCCCATG	Conserved	miR-1388	√	√

miR _6	GGCTTGTTTTAAGTTGCCTGCG	Conserved	miR-1788	√	√

miR _7	TTACAGGCTATGCTAATCTATG	Non-conserved	-	√	No

miR _8	AAGGTCCAACCTCACATGTCC	Non-conserved	-	√	No

miR _9	TGATTGTTTGTATCAGCTGTGT	Non-conserved	-	√	No

miR _10	TAGGGGTATGATTCTCGC	Non-conserved	-	√	No

miR _11	TAGGTAGTTTGATGTTGTTGGG	Conserved	miR-196	√	√

miR _12	CGGCCCGTCCGGTGCGCTCGGAT	Non-conserved	-	√	No

miR _13	TCACACCTACAATCCCTGGCA	Non-conserved	-	√	No

miR _14	AAAGTGAAAGGTGACTGAGAC	Non-conserved	-	√	No

miR _15	TAGGTAGTTTTATGTTGTTGGG	Conserved	miR-196	√	√

miR _16	AGCAGCATTGTACAGGGCTTT	Conserved	miR-107	√	√

miR _17	GTATGTGCCCTTGGACTACATT	Conserved	miR-455	No	√

miR _18	TATGTGTGTATCAATTGTGTGAAA	Non-conserved	-	√	No

miR_19	GTAATGCTTCGACTGATTGGTG	Non-conserved	-	√	No

miR _20	AGATTGGGGTGAGTTAGGGTG	Non-conserved	-	√	No

miR _21	AGCTACATCTGAATACTGGGTCA	Conserved	miR-222	√	√

miR _22	CCTCTCTGTGCTGCCATTTGGGAC	Non-conserved	-	√	No

miR _23	ATGATTCGACTCATATGGTG	Non-conserved	-	√	No

miR _24	AGCTCGTGTCCCAAGGCGCCT	Non-conserved	-	√	No

miR_25	TCGTACCGTGAGTAATAGTGCA	Conserved	miR-126	No	√

The novel miRNAs were numbered sequentially from 1 to 25.

**Figure 6:**
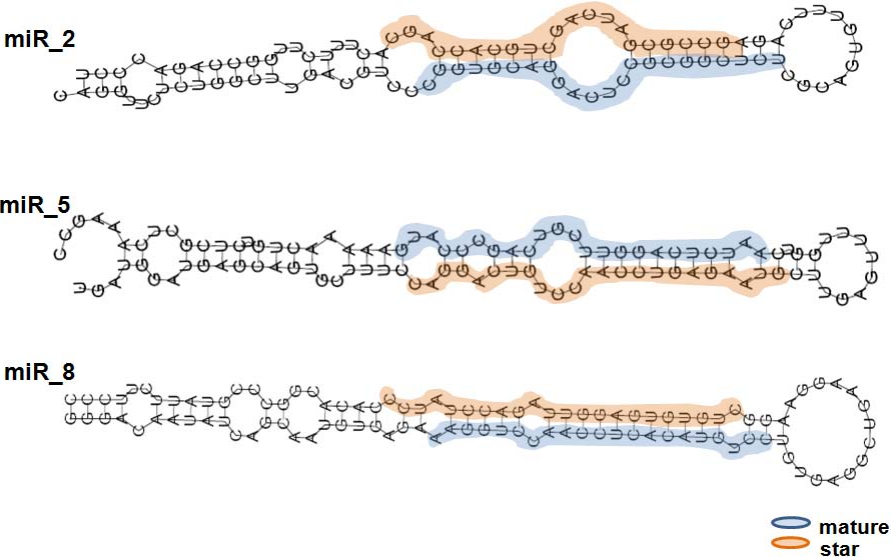
**RNA secondary structure of the novel miRNAs**. The secondary structures were retrieved by RNAfold, which is included in the mirDeep software package. Both mature and star sequences of the novel miRNAs are indicated. The mature sequence is highlighted in blue and the star sequence is in orange. The structures were drawn from the 5' end to the 3' end. The structures of miR_2, miR_5 and miR_8 are shown to exemplify the protocol used to identify the ZF miRNAs.

Star sequences were also identified for at least five of the novel miRNAs, supporting the authenticity of the corresponding miRNAs, as their detection is an important criterion for miRNA validation (Figure 6). Such star sequences are small RNAs complementary to mature miRNAs, which are produced during pre-miRNA processing, but are not loaded into the RISC complex and are degraded [1]. Indeed, star sequences were not identified for 20 novel low abundance miRNAs, most likely due to their rapid turnover. Most of the new miRNA genes were intergenic rather than intronic, however the novel highly expressed miR_8 was non-conserved and intronic (see Additional File 4). This miRNA was found within the coding region of the *ANK1 *gene, which codes for an intracellular protein required for biogenesis and maintenance of membrane domains in both excitable and non-excitable cells in diverse tissues, namely erythrocyte, kidney, lung and brain [54]. Some of the novel miRNAs were encoded by more than one gene. For example, miR_2 was encoded by 4 different genes and miR_10, miR_12, miR_18 and miR_22 were encoded by 2 different genes, as determined by miRDeep.

The expression of a conserved miRNA (miR_4), a non-conserved miRNA for which the star sequence was also detected (miR_8) and a non-conserved miRNA for which the star sequence was not detected (miR_9), was validated by qRT-PCR (Figure 5B). As above, there was strong correlation between qRT-PCR and 454 pyrosequencing data. Indeed, miR_8 displayed the highest number of pyrosequencing reads and had higher relative abundance in the qRT-PCR analysis. Also, miR_4 and miR_9 had similar levels of expression, but lower than miR_8.

### Target prediction for novel miRNAs

The miRNA targets can be predicted computationally with high confidence for conserved miRNAs, but such predictions remain challenging for non-conserved miRNAs due to restrictions imposed by the search algorithms used in the target prediction databases [55]. For the non-conserved miRNAs only the more extensively paired interactions can be predicted with reasonable confidence. In order to minimize noise (false predictions) in the prediction of targets, stringent criteria similar to those described by Sunkar and colleagues [11] were used. This was based on blast searches for antisense hits with less than 6 mismatches, with perfect seed match and thermodynamic stability using the *RNA HYBRID *software. Forty two putative gene targets of the 16 newly identified miRNAs, which were mainly involved in binding nucleotides, proteins or ions or had catalytic activity, were identified (see Additional File 5). The predominant biological functions of these predicted target genes were cellular processes related to metabolism and signal transduction and developmental processes, including embryonic patterning, vasculogenesis and neuron differentiation. Most miRNAs with predicted targets involved in developmental processes were detected in cDNA libraries prepared from miRNA samples collected during embryonic development. For example, miR_7 was detected at 5 dpf; miR_8 was detected at 72 hpf, 96 hpf and 5 dpf; miR_9 and miR_10 were both detected at 72 hpf and 96 hpf; miR_16 was detected at 72 hpf and 5 dpf. The *MELK *gene which is found in gills and is involved in erythrocyte development was predicted to be targeted by miRNA_14 raising the hypothesis that miR_14 could be involved in erythrocyte development [56]. Other possible targets encoded hypothetical proteins and, for this reason, were not included in our analysis. This approach was unable to identify candidate targets for some of the novel miRNAs, a result that may be explained by the high stringency of the prediction algorithm because targets of conserved miR_4 and miR_11 were also missed. Alternatively, some of these low abundance non-conserved miRNAs may have appeared recently and do not yet have targets, or misannotation or incomplete annotation of the ZF genome may have prevented identification of such targets.

### Star sequences of zebrafish microRNAs

**Figure 7:**
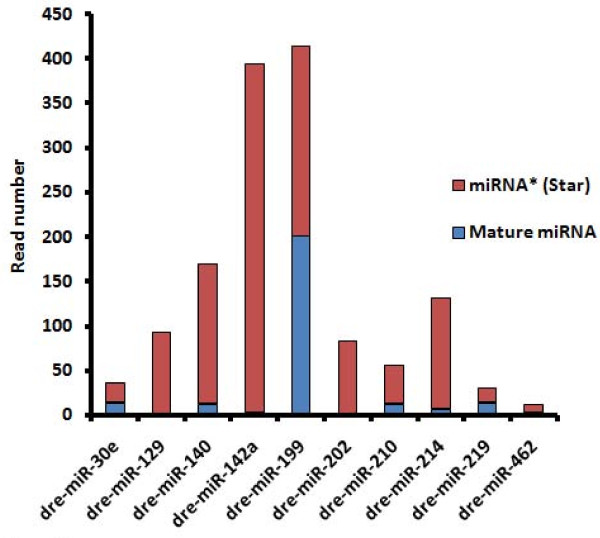
**Zebrafish miRNA star sequences**. In some ZF samples, the number of miRNA* reads was higher than that of mature miRNA sequences, namely dre-miR-129*, dre-miR-140*, dre-miR-142a*, dre-miR-202*, dre-miR-210* and dre-miR-214*. Dre-miR-142a* had the highest number of sequence reads (391). Dre-miR-30e, dre-miR-199, dre-miR-219 and dre-miR-462 showed similar strand-bias towards both mature and star strands, suggesting that both strands may be incorporated into the RISC complex.

Star sequences for 50% of the miRNAs, both known and novel, were also detected and identified (see Additional File 2). This corresponded to 102 star sequences complementary to known miRNAs. Of these, 42 were sequenced previously [19,20] or registered in miRBase and 5 were complementary to the novel miRNAs. As expected, the majority of reads corresponded to annotated miRNAs rather than to miRNAs*. This was in agreement with the miRNA biogenesis model and resulted from incorporation into the RISC complex and protection from degradation of the strand with lower 5' end thermodynamic stability [7]. However, in some cases, the number of miRNA* reads was similar or even higher than that of the corresponding mature miRNAs (Figure 7). This could be explained by similar 5' end stability of miRNA and miRNA* strands and similar incorporation efficiency into the RISC complex. For example, dre-miR-30e, dre-miR-199, dre-miR-219 and dre-miR-462 showed similar bias for both mature and star strands. In the case of dre-miR-129, dre-miR-140, dre-miR-142a, dre-miR-202, dre-miR-210 and dre-miR-214, the number of miRNA* reads was considerably higher than that of mature miRNA reads, suggesting that the miRNA* strand was more stable than the miRNA strand. Therefore, it is likely that some miRNAs* acquired mRNA targets and could also regulate gene expression in ZF [57]. Quantification of miRNA and miRNA* expression by qRT-PCR (Figure 5C) confirmed the higher relative abundance of miR-140* and miR-142a* and showed that dre-miR-142* was expressed during development while its mature miRNA was not detected. The relative abundance of miR-199* and miR-199 determined by qRT-PCR further confirmed the pyrosequencing data (Figure 5C).

## Discussion

To date, 192 ZF miRNAs have been identified using classical cloning and Sanger sequencing methodologies [19,20]. In this study, 25 novel miRNAs were added to the ZF repertoire using massively parallel DNA pyrosequencing of miRNA cDNA libraries prepared from different time points of the ZF embryo development and from different tissues. This methodology retrieved 173 of the 192 known ZF miRNAs whose expression in different tissues and developmental stages were validated using Northern blot analysis and/or *in situ *hybridizations. This high degree of data overlapping between cloning/Sanger sequencing and DNA pyrosequencing, plus the existence of target genes for the novel miRNAs, validated our approach of miRNA libraries fractionation and provided strong support for the authenticity of the newly identified miRNAs. The pyrosequencing approach also produced important information about the relative abundance of the ZF miRNAs. During early development (24 hpf), the number of different miRNAs was low (13), but expression level was high. The number of miRNA reads was higher at 72 hpf and in young adult fish. Among differentiated organs, brain and eyes showed the highest number of miRNA reads. This confirmed previous data showing differences in temporal miRNA expression and raised the hypothesis that many miRNAs play a role in late development and are required for organ morphogenesis [20].

### Zebrafish microRNAs expression profile

The pyrosequencing data allowed us to build a miRNA expression profile for developmental differentiation and for adult fish, based on the normalized number of reads. Most miRNAs were expressed in more than one tissue (Figure 4A), others were tissue specific or showed stronger expression in specific tissues (Figure 4B), while others were development specific. Dre-miR-135c and dre-miR-25 were highly enriched at 24 hpf, but their relative expression decreased during embryo development. The data confirmed previous studies showing that dre-miR-135 expression is higher in development than in adult fish [20]. The miR-430 family was also present during development and was absent in adult ZF [19,20]. The expression profile also highlighted results of Giraldez and colleagues [39] showing that miR-430 is essential for regulation of morphogenesis during development.

Some miRNAs were expressed ubiquitously. For example, dre-miR-124 was abundant during both development and in adult fish, and its expression increased slightly during late stages of development and in the central nervous system (both brain and eyes). This miRNA alone accounted for ~48% of the pyrosequencing reads, a result that may be explained, at least in part, by the high copy number of its gene (6 copies in various chromosomes). At 24 hpf, when a significant part of the brain development had already occurred, dre-miR-124 represented 42% of the miRNA pool and its relative abundance reached 80% at 5 dpf. In the adult tissues, it represented 80% of brain and 54% of eye miRNAs. This is in agreement with previous studies in ZF and other organisms showing that miR-124 is up-regulated during development of the nervous system and is the most abundant miRNA in the adult brain [19,20]. Also, neuronal differentiation is enhanced followed transfection of mir-124 in mouse neuronal stem cells [35]. Taken together, the data suggest that dre-miR-124 may play an important role in ZF development, neuronal differentiation and in regulation of brain functions [35,58]. On the other hand, dre-miR-203a and dre-miR-203b appeared early in development and maintained high levels of expression in adult fish, in particular in gills and skin. Indeed, miR-203 is a skin-specific keratinocyte-derived miRNA involved in keratinocyte differentiation [59].

A subset of miRNAs was expressed in differentiated tissues only. For example, dre-miR-101a, dre-miR-130b, dre-miR-130c, dre-miR-221 and dre-miR-499 were highly enriched in the heart, in agreement with previous *in situ *and Northern blot studies [20]. Dre-miR-1 and dre-miR-133a were expressed in muscle and heart, where they play an important regulatory role in other organisms [60,61]. Indeed, deletion of miR-1 altered regulation of cardiogenesis, electrical conduction and cell cycle of cardiomyocites, and miR-133 plus miR-1 regulate cardiac hypertrophy, as their over expression inhibits it. Interestingly, dre-miR-133b and dre-miR-133c were mainly detected in muscle and were not present in the heart. Finally, dre-miR-103 was specific of gut and liver while dre-miR-122 was liver specific [40,62]. This was not surprising because mir-122 plays important roles in regulation of metabolism and its silencing in high-fat fed mice resulted in a significant reduction of hepatic steatosis, decreased cholesterol synthesis and stimulated fatty-acid oxidation [63].

### Expression and putative functions of the novel zebrafish miRNAs

Of the 25 novel miRNAs identified in this study, 9 belong to conserved miRNA families (existing in at least one more organism) according to the conservation criteria used in this study, and the others are non-conserved (ZF specific). Most of the novel miRNAs are encoded by a single gene, but 7 are multigenic. In the latter case, miRDeep, Ensembl and RNAfold analysis showed that different genes encoding a single miRNA produce identical miRNA hairpins. Most of the novel miRNAs produced lower number of reads than the majority of the conserved miRNAs. This was not surprising since there is good correlation between miRNA conservation and expression level [64]. Therefore, the low abundance of the novel miRNAs identified by pyrosequencing combined with the retrieval of 90% of the known ZF miRNAs (identified by cloning/Sanger sequencing) suggests that the vast majority of miRNAs present in our samples were retrieved. However, one cannot exclude the hypothesis that new miRNAs present in our dataset escaped identification due to the high stringency of the methodology used. Also, it is possible that other low abundance and highly specific ZF miRNAs may still be discovered using other deep DNA pyrosequencing strategies, namely Solexa/Illumina or SOLiD™. Finally, cDNA libraries from tissues not screened in this study may still reveal new ZF miRNAs. Recent bioinformatics analysis of the ZF genome identified additional miRNAs [44], however we were unable to identify reads matching these putative miRNAs using miRDeep alone or our pipeline data analysis system. This may be due to their very low expression level. Again, other massively parallel DNA pyrosequencing approaches may overcome these limitations and uncover such putative miRNAs [44].

Our bioinformatics approach retrieved 41 candidate target genes of 15 novel miRNAs. Since we used stringent search criteria to minimize false positives one cannot exclude the possibility that some targets were missed. Despite this, comparative analysis of the targets of the conserved miR_15, miR_16 and miR_21 with those of known miRNAs produced significant overlapping, thus validating our target prediction approach. For example, miR_16, which belongs to the miR-107 family, has *GFM2 *and *VOX *genes as putative targets. The miRBase Targets Version 5 also retrieved these genes as candidate targets for dre-miR-107. Similar results were obtained for miR_21 where *RNF11 *gene was highlighted as a candidate target of this novel miRNA of the mir-222 family. This result is also supported by retrieval of miR-222 in a blast search for miRNAs that target *RNF11*.

Most of the predicted targets are involved in cellular and developmental processes, which is in agreement with their expression during development. Indeed, the *NRP2A *gene, a putative target of miR_8, is involved in the differentiation of the nervous system, neural crest cell migration [65] and in VEGF-mediated vessel development [66]. This correlates with the expression pattern of this miRNA at 72 hpf, 96 hpf, 5 dpf and in the adult brain. Also, miR_9, expressed at 72 hpf and 96 hpf, is predicted to target the *PRDM1 *and *zgc:85707 *genes which play important roles in embryonic axis specification, embryonic pectoral fin morphogenesis, regulation of neuron specification, regulation of signal transduction and multicellular organism development [67]. The *SEC23B *and *MYST3 *genes which are involved in cartilage development were retrieved as putative targets of miR_10, which was also expressed during development. *MYST3 *(or *MOX*) regulates *HOX *expression and segmental identity, including cartilage patterning [68]. Finally, the gills specific miR_14 is predicted to target the *MELK *gene, which is also strongly expressed in the gills and is involved in erythrocyte development [56].

### Zebrafish microRNA star sequences

Star sequences of miRNAs (miRNA*) are difficult to detect by conventional methods due to their rapid turnover. However, high throughput sequencing retrieved many of them and revealed their relative abundance in different organisms [19,20,57,64]. Our DNA pyrosequencing approach identified 107 miRNA* sequences: 42 were identified previously by cloning and Sanger sequencing [19,20], 60 were identified in this pyrosequencing study, but belong to known miRNAs, and other 5 miRNA* belong to the novel miRNAs identified in this study. Most star sequences retrieved fewer reads than the corresponding mature miRNAs which is consistent with the miRNA biogenesis model and strand selection by RISC. However, six miRNA* were more abundant than their corresponding mature miRNAs, namely dre-miR-129*, dre-miR-140*, dre-miR-142a*, dre-miR-202*, dre-miR-210* and dre-miR-214*. Similar results were observed before for dre-mir-129*, dre-mir-142a*, dre-mir-142b* and dre-mir-214* [20]. Dre-miR-30e, dre-miR-199, dre-miR-219 and dre-miR-462 showed similar strand-bias of both mature and star strands. Since this was also observed in the chicken embryo for mir-30e and mir-219 [64] and in *Drosophila melanogaster *where several miRNA* are present at physiologically relevant levels and associate with Argonaute proteins, it is likely that both strands are loaded into RISC and may guide target repression. Finally, observed alterations in the ratio of expression of mature/star molecules suggests that some star molecules are functional and their activity may vary according to cellular context [57,64]. Obviously, the biological function of these star sequences can only be unravelled through experimental testing, but their high number of reads suggests their inclusion in future ZF miRNA chips and expression profiling studies.

## Conclusion

This study increased the total number of ZF miRNAs from 192 to 217 and showed that miRNA cDNA libraries prepared from different developmental stages and from adult tissues is an effective methodology for miRNA discovery using low cost DNA pyrosequencing mini-chips. The methodology permitted quantitative and qualitative analysis of miRNA expression throughout the ZF life cycle, as the miRNA profile was largely in agreement with qRT-PCR and Sanger sequencing data. Most of the 25 novel miRNAs were non-conserved low abundance molecules and their targets indicated that they might be involved in developmental processes. Novel miRNA star sequences were also identified and some of them were more abundant than their corresponding mature miRNAs, suggesting that they may also be loaded into RISC and may be functional. Future deep sequencing studies may still identify additional miRNAs in ZF, however such miRNAs may be expressed at very low levels or in specific physiological or pathological conditions.

## Methods

### MicroRNA library construction and sequencing

Small RNA libraries were prepared from different ZF developmental stages, namely 24 hours post-fertilization (hpf), 72 hpf, 96 hpf, 5 days post-fertilization (dpf), 45 dpf, young adult and from adult brain, eyes, gills, muscle, heart, skin, fins, and gut/liver (Figure 1). Briefly, 100 μg of total RNA from each sample was isolated using TRIzol^® ^and small RNAs were enriched by differential precipitation using polyethylene glycol. Total RNAs were fractioned using 12% denaturing PAGE and small RNAs of 15–30 nt were gel-isolated using Gel Filtration cartridges from Edge Biosystems. For cDNA synthesis, the small RNA molecules previously isolated were first ligated to a 3' adapter (AMP-5'p-5'p/CTGTAGGCACCATCAATdi-deoxyC- 3') in absence of ATP and gel excised in the range of 35 and 50 nt. A second ligation was performed with the 5' adapter ("Nelson's linker" 5'ATCGTrArGrGrCrArCrCrUrGrArArA 3'), for 1 hour at 37°C, followed by phenol extraction. First strand cDNA synthesis was then performed using a specific 3'-primer and Superscript™ III reverse transcriptase (Invitrogen). RNAseH treated cDNA was PCR-amplified with adapter specific primers. PCR products were then run on 10% denaturing PAGE containing 7 M urea and the corresponding band (100 nt) was eluted from the gel with Probe Elution Buffer from Ambion, at 37°C overnight. Parallel DNA pyrosequencing was performed using the Genome Sequencer FLX (Roche), following established protocols for DNA library sequencing [42].

### Computational analysis of sequencing reads

Base calling and quality trimming of sequence reads was carried out using the Genome Sequencer FLX software. Raw images were processed to remove background noise and the data was normalized. TAGs and adapter sequences of ZF developmental and adult tissues samples were then identified and trimmed and those reads with correct TAGs and adapters (> 15 nt) were retrieved for downstream analysis using the miRDeep software , with a cut-off value of 1. Initially, miRDeep aligned the sequences against the zebrafish genome using megaBlast with seed length set at 12, the traditional blast output, and minimum local identity set at 100. The blast output was then parsed for miRDeep uploading and aligned sequences with a maximum of 2 mismatches in the 3' end were retrieved. Reads that matched more than 10 different genome loci were discarded and only those with one or more alignments were kept and, using the remaining alignments as guidelines, the potential precursors were excised from the genome. The secondary structure of putative precursors was predicted using RNAfold and signatures were created by retaining reads that aligned perfectly with those putative precursors to generate the signature format. Finally, miRDeep predicted miRNAs by discarding non-plausible Dicer products and scoring plausible ones. To assess seed conservation, plausible Dicer processing sequences were blasted against a local version of mature miRNAs from miRBase 12.0 that lacked zebrafish miRNA sequences. Borderline miRNA candidates were also resolved by determining their relative stability using Randfold. To distinguish between novel and known miRNAs, selected pre-miRNAs were blasted against *Danio rerio *stem loop sequences (miRBase) and those that did not produce any or produced perfect alignments were scored as novel miRNAs. Pairs of signatures and structures were used to estimate the number of false positives by randomly permutating them, using miRDeep.

To estimate the false negative rate, known mature miRNAs deposited in miRBase 12.0 were used to carry out a megaBlast search using our sequencing data set. Perfect alignments were considered as true positives. This list of miRNAs was then compared with that of the miRNAs predicted by miRDeep and the sequences present in the blast list but absent in the miRDeep list were considered false negatives. To overcome the inherent lack of sensitivity of miRDeep, novel transcripts encoding miRNAs predicted by bioinformatics were retrieved from Ensembl 5.2 using BioMart and from literature predictions [43]. These sequences were then used to perform a megaBlast search against our data with seed length set at 12. The transcripts with perfect matches and alignment length larger than 18 nucleotides were kept for further processing. These transcripts were then compared with the mature miRNAs present in miRBase 12.0 and those that produced imperfect alignments or did not produce alignments were considered new miRNAs.

Reads without matches in the ZF genome (megaBlast) were re-aligned using SHRiMP,  which handles short reads. Alignments were carried out using the space seed 011111111000; where 1 is the number of seed matches per window. Suboptimal alignments were retrieved and transformed into the blastparsed format for miRDeep miRNA prediction.

### Conservation assessment

Novel microRNAs were considered conserved whenever they showed > 90% identity for the mature sequence and > 60% identity for the precursor, as in previous studies [20,52].

### Statistical analysis of miRNA population

A rarefaction analysis of the detected miRNA population was carried out to assess the representativeness of the miRNA reads. A rarefaction curve, of the total number of reads obtained *vs *the total number of miRNA species, was plotted. The non-parametric richness estimator, Chao1 [49] was determined to predict the total richness of the miRNA population, as a function of the observed richness (*S*_*obs*_), the number of sequences observed only once (singletons, *n*_*1*_) and the number of sequences observed twice (doubletons, *n*_*2*_). A file containing the total number of reads of each miRNA was generated and used as input data set for the EstimateS8.0 statistical package [48]. Both the rarefaction curve and the Chao1 statistical estimator were computed using EstimateS8.0.

### Zebrafish miRNA expression profile

Read numbers were normalized as described by Chen and colleagues [19] and a miRNA expression profile, using identical number of reads for each sample was generated. The number of reads between samples was normalized as indicated below:



Where NRmiRNA_X_^Y ^is the number of reads of miRNA_X _(X = any miRNA) in sample Y, and TNRmiRNAs^Y ^is the total number of miRNAs in sample Y. 1000 is an arbitrary number of reads. The data was transformed into log2 scale to build the heat map using the MeV 4.0 software package .

### MicroRNA expression analysis by quantitative Real-Time PCR

miRNA expression was quantified using the NCode™ SYBR^® ^Green miRNA RT-PCR Kit (InVitrogen) according to the manufacturers' instructions. One microgram of total RNA was used for cDNA synthesis. Reverse transcriptions were carried out in triplicate and analyzed using a 7500 Real-Time PCR System (Applied Biosystems). A dissociation curve was generated at the end of each PCR cycle to check for single product amplification. Quantification of target cDNA was determined by converting Ct values to cDNA copy number using the following equation:



where Ct(sc) is the Ct expected for a sample containing a single copy template and E is the PCR efficiency. All Ct values above 35 were set to 35 and a PCR efficiency of 0.90 and Ct(sc) = 35 were assumed [69].

### Target predictions

The 3'UTR sequences of ZF mRNAs were extracted from Biomart  and blasted against the antisense miRNA sequence for the new miRNAs or against the antisense miRNA* sequence, in the case of the star sequences. Sequences with perfect seed match between nucleotides 2 and 7, and no more than 6 mismatches in the remaining sequence, were retained for further analysis. Targets were considered positive whenever RNAhybrid confirmed them thermodynamically. Targets were discarded when RNAhybrid did not retrieve the targets obtained in the first approach.

## Abbreviations

ZF: zebrafish; 3' UTR: 3' Untranslated Region; RISC: RNA induced silencing complex; miRNA: microRNA; hpf: hours post fertilization; dpf: days post fertilization; GO: Gene ontology.

## Competing interests

The authors declare that they have no competing interests.

## Authors' contributions

ARS, PMP and MASS conceived and design the study, ARS and PMP prepared miRNA samples and cDNAs, CE carried out pyrosequencing of the cDNA libraries, BS and ARS did the bioinformatics analysis of sequencing results. JA and JLO extracted the 3'UTRs from ZF mRNAs and did the blast analysis. ARS did the targets predictions, data analysis and wrote the manuscript. ACG did the Chao1 analysis, GM, MASS and PMP supervised the study and corrected the manuscript. All authors read and approved the final manuscript.

## Supplementary Material

Additional file 1 **Data pipeline analysis**. Scheme representing the data pipeline used to analyze pyrosequencing data. **(A) **includes miRDeep analysis and **(B) **further processing of miRDeep discarded reads. Click here for file

Additional file 2 List of known miRNAs and miRNA*. List of known miRNAs and miRNA* identified in this study and its distribution in the small RNA libraries. Click here for file

Additional file 3 **Number of miRNA reads and unique miRNAs throughout development (A) and mature tissues (B)**. Correlation between the numbers of miRNA reads for each tagged sample and the number of unique miRNAs of each sample. Click here for file

Additional file 4 **Putative novel miRNAs**. List of the novel miRNAs identified, together with information about genomic location, conservation, expression, number of reads and presence or absence of a star sequence. Click here for file

Additional file 5 **Targets for novel miRNAs**. List of the genes that are targets of the novel miRNAs and their classification by GO terms. Click here for file
