# Supplementary material for: Parallel DNA pyrosequencing unveils new zebrafish microRNAs
Source: BMC Genomics. 2009 Apr 27;10:195. doi: 10.1186/1471-2164-10-195 (PMC2684549; doi:10.1186/1471-2164-10-195)
Supplement: Additional file 1 — Data pipeline analysis. Scheme representing the data pipeline used to analyze pyrosequencing data. (A) includes miRDeep analysis and (B) further processing of miRDeep discarded reads. [file 1471-2164-10-195-S1.pdf]

Additional File 1, Figure A1 – Data pipeline analysis.

Flowchart describing the procedure used for identify microRNAs in pyrosequencing data. **(A)** includes miRDeep analysis and **(B)** further processing of miRDeep discarded reads. The red boxes stand for data coming from databases. -i and -d stands for query file and database respectively.

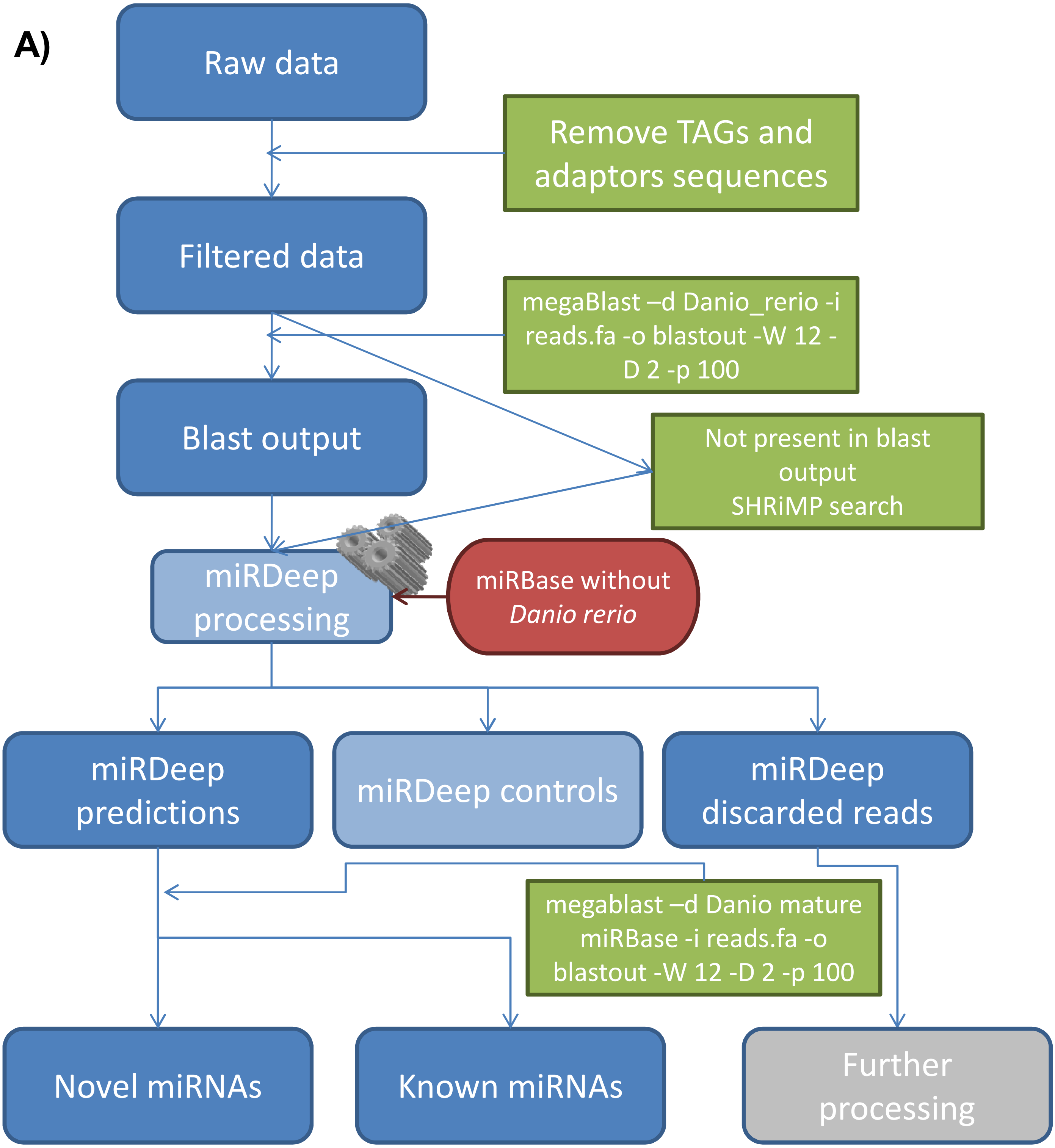

B)

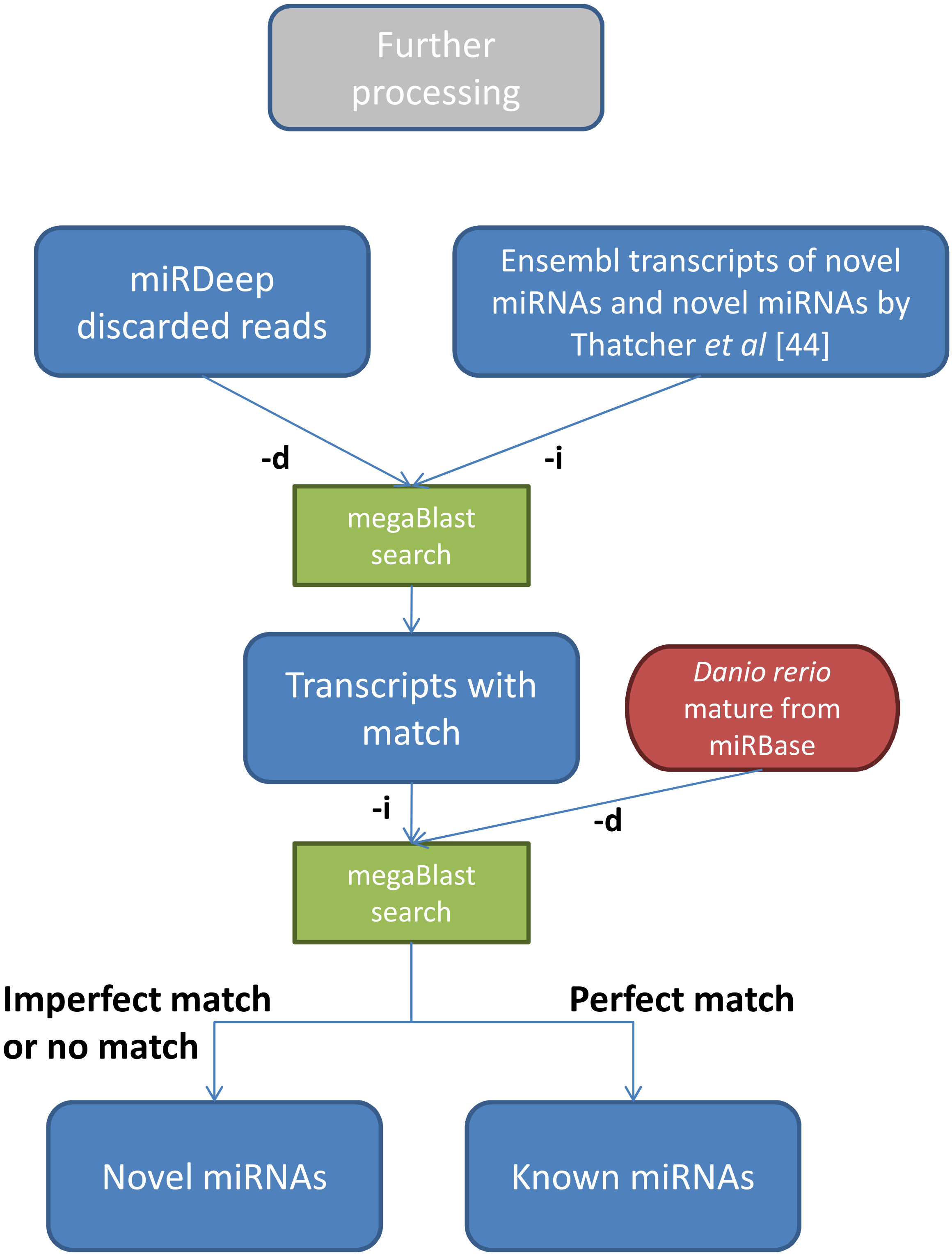
Further processing
miRDeep discarded reads
Ensembl transcripts of novel miRNAs and novel miRNAs by Thatcher et al [44]
-d
-i
megaBlast search
Transcripts with match
Danio rerio mature from miRBase
-i
-d
megaBlast search
Imperfect match or no match
Perfect match
Novel miRNAs
Known miRNAs
