# Supplementary material for: Parallel DNA pyrosequencing unveils new zebrafish microRNAs
Source: BMC Genomics. 2009 Apr 27;10:195. doi: 10.1186/1471-2164-10-195 (PMC2684549; doi:10.1186/1471-2164-10-195)
Supplement: Additional file 3 — Number of miRNA reads and unique miRNAs throughout development (A) and mature tissues (B). Correlation between the numbers of miRNA reads for each tagged sample and the number of unique miRNAs of each sample. [file 1471-2164-10-195-S3.doc]

### Additional file 3 – Number of miRNA reads and unique miRNAs throughout development (A) and adult tissues (B).

A)

| **Developmental stage** | **Nr of reads** | **Nr unique miRNAs** |
| --- | --- | --- |
| **24hpf** | 27 | 13 |
| **72hpf** | 6786 | 149 |
| **96hpf** | 1385 | 91 |
| **5dpf** | 2166 | 90 |
| **45dpf** | 1603 | 79 |
| **Adult** | 5715 | 146 |

B)

| **Adult Tissue** | **Nr of reads** | **Nr unique miRNAs** |
| --- | --- | --- |
| **Brain** | 9825 | 160 |
| **Eyes** | 4099 | 103 |
| **Heart** | 401 | 65 |
| **Gills** | 2285 | 107 |
| **Muscle** | 600 | 76 |
| **Fins** | 981 | 82 |
| **Skin** | 222 | 58 |
| **Gut/Liver** | 571 | 55 |

The number of miRNA reads for each tagged sample was calculated and correlated with the number of unique miRNAs of each sample. The tables show those measurements, for both the development samples and tissue samples.
