# Supplementary material for: Parallel DNA pyrosequencing unveils new zebrafish microRNAs
Source: BMC Genomics. 2009 Apr 27;10:195. doi: 10.1186/1471-2164-10-195 (PMC2684549; doi:10.1186/1471-2164-10-195)
Supplement: Additional file 4 — Putative novel miRNAs. List of the novel miRNAs identified, together with information about genomic location, conservation, expression, number of reads and presence or absence of a star sequence. [file 1471-2164-10-195-S4.doc]

## Additional File 4 - Putative novel miRNAs

| **miRNA ID** | **miRNA sequence** | **Genomic Location** |  | **Conservation** | **Expression** | **Nº Reads** | **Star sequence found** |
| --- | --- | --- | --- | --- | --- | --- | --- |
| **miR_1** | AACAGTAAGAGTTTATGTGCTG | Chr. 10 | Intergenic | Non-conserved | Gills | 41 | No |
| **miR_2** | CGGTGCAGGACTCCGCGGCTC | Zv7_NA4058  Chr. 1  Chr. 5  Chr. 17 | Intronic  Intronic  Intronic  Intronic | Non-conserved | Fins, Heart | 3 | Yes |
| **miR_3** | AAGTGGCCTCTAAAAGTCTA | Chr.12 | Intergenic | Non-conserved | Gills | 1 | No |
| **miR_4** | TAATACTGCCTGGTAATGCCAT | Chr.6 | Intergenic | Conserved | 72h, 96h,  5d, Gills,  Eyes, Fins | 27 | No |
| **miR_5** | ATCTCAGGTTCGTCAGCCCATG | Chr. 23 | Intergenic | Conserved | 5d, Brain,  Fins, Muscle | 7 | Yes |
| **miR_6** | GGCTTGTTTTAAGTTGCCTGCG | Chr. 9 | Intergenic | Conserved | 72h, Gills,  Fins,  Eyes | 26 | Yes |
| **miR_7** | TTACAGGCTATGCTAATCTATG | Chr. 17 | Intergenic | Non-conserved | 5d, Brain | 5 | Yes |
| **miR_8** | AAGGTCCAACCTCACATGTCC | Chr.5 | Intronic | Non-conserved | 72h, 96h,  5d,Brain,  Eyes, Skin,  Gills | 149 | Yes |
| **miR_9** | TGATTGTTTGTATCAGCTGTGT | Chr.9 | Intergenic | Non-conserved | 72h, 96h, Adult | 14 | No |
| **miR_10** | TAGGGGTATGATTCTCGC | Zv7_scaffold2553  Zv7_scaffold2559 | Intergenic  Intergenic | Non-conserved | 72h, 96h, Adult | 12 | No |
| **miR_11** | TAGGTAGTTTGATGTTGTTGGG | Chr. 11 | Intergenic | Conserved | 72h, 96h | 4 | No |
| **miR_12** | CGGCCCGTCCGGTGCGCTCGGAT | Chr. 7  Chr. 5 | Intergenic  Intergenic | Non-conserved | Adult | 8 | No |
| **miR_13** | TCACACCTACAATCCCTGGCA | Chr. 17 | Intergenic | Non-conserved | Brain | 2 | No |
| **miR_14** | AAAGTGAAAGGTGACTGAGAC | Chr. 12 | Intronic | Non-conserved | Gills | 2 | No |
| **miR_15** | TAGGTAGTTTTATGTTGTTGGG | Chr. 16 | Intergenic | Conserved | 72h | 1 | No |
| **miR_16** | AGCAGCATTGTACAGGGCTTT | Chr. 17 | Intronic | Conserved | 72h, 5d, Gills | 4 | No |
| **miR_17** | GTATGTGCCCTTGGACTACATT | Chr. 21 | Intronic | Conserved | Brain | 2 | No |
| **miR_18** | TATGTGTGTATCAATTGTGTGAAA | Chr. 20  Chr. 7 | Intergenic  Intergenic | Non-conserved | 72h | 1 | No |
| **miR_19** | GTAATGCTTCGACTGATTGGTG | Chr. 22 | Intergenic | Non-conserved | Gills | 1 | No |
| **miR_20** | AGATTGGGGTGAGTTAGGGTG | Chr.3 | Intergenic | Non-conserved | Gills | 1 | No |
| **miR_21** | AGCTACATCTGAATACTGGGTCA | Zv7_NA1292 | Intronic | Conserved | Gills | 3 | No |
| **miR_22** | CCTCTCTGTGCTGCCATTTGGGAC | Chr.12  Zv7_NA2627 | Intergenic  Intergenic | Non-conserved | Gills | 1 | No |
| **miR_23** | ATGATTCGACTCATATGGTG | Chr.1 | Intergenic | Non-conserved | Gills | 3 | No |
| **miR_24** | AGCTCGTGTCCCAAGGCGCCT | Chr.2 | Intergenic | Non-conserved | Brain | 3 | No |
| **miR_25** | TCGTACCGTGAGTAATAGTGCA | Chr. 10 | Intronic | Conserved | Brain | 2 | No |

A list of the novel miRNAs identified is given, together with information about genomic location, conservation, expression, number of reads and presence or absence of a star sequence.
