# Supplementary material for: Parallel DNA pyrosequencing unveils new zebrafish microRNAs
Source: BMC Genomics. 2009 Apr 27;10:195. doi: 10.1186/1471-2164-10-195 (PMC2684549; doi:10.1186/1471-2164-10-195)
Supplement: Additional file 5 — Targets for novel miRNAs. List of the genes that are targets of the novel miRNAs and their classification by GO terms. [file 1471-2164-10-195-S5.doc]

## Additional File 5 - Targets for novel miRNAs

| **miRNA ID** | **Gene ID**  **(ZFIN)** | **Gene name** | **Match** | **Mfe (kcal/mol)** | **GO**  **Molecular Function** | **GO**  **Biological process** | **GO**  **Cellular component** |
| --- | --- | --- | --- | --- | --- | --- | --- |
| **mir_3** | zgc:76917 | TIA1 cytotoxic granule-associated RNA binding protein 1 | target 5' U UU UUU G 3'  UUGU GU UAGGCCCCAG  AACG CA AUCCGGGGUC  miRNA 3' U U 5' | -29,1 | binding: nucleotide | unknown | unknown |
| **mir_3** | zgc:63722 | phosphoglycerate mutase 1 like b | target 5' A AA U U A 3'  UUG UAU G GCCCCAG  AAC AUA C CGGGGUC  miRNA 3' GUC U 5' | -21 | catalytic activity | cellular process : glicolysis | Unknown |
| **mir_3** | ss18 | synovial sarcoma translocation | target 5' C CCC UU U 3'  UGC AGUGUG UCCCAG  ACG UCAUAU GGGGUC  miRNA 3' A CC 5' | -21,6 | unknown | unknown | unknown |
| **mir_3** | vasn | Vasorin | target 5' U AC A 3'  CAG UGGGCCCCA  GUC AUCCGGGGU  miRNA 3' AAC AU C 5' | -25,4 | binding: DNA | unknown | unkown |
| **mir_5** | rap2ip | Rap2 interacting protein | target 5' C CA U C 3'  UAUG CUGAC GACCUGAGA  GUAC GACUG UUGGACUCU  miRNA 3' CC C A 5' | -29,7 | unknown | unknown | unknown |
| **mir_7** | kdrb | Vascular endothelial growth factor receptor 2 precursor (VEGF2) | target 5' G UA UU C 3'  AGAUU CAU CCUGUA  UCUAA GUA GGACAU  miRNA 3' GUA UC UC U 5' | -19,3 | binding: nucleotide | development process: angiogenesis | signal recognition particle |
| **mir_8** | nrp2a | Neuropilin 2a | target 5' G G C U CC C 3'  GGA AU UG GG GGACCUU  CCU UA AC CC CCUGGAA  miRNA 3' G C U AA 5' | -25,0 | receptor activity | developmental process: vasculogenesis | membrane |
| **mir_9** | prdm1 | Blimp-1 homolog | target 5' U AA A U 3'  ACACA UGAUA AGACAAUCA  UGUGU ACUAU UUUGUUAGU  miRNA 3' CG G 5' | -26,2 | binding: nucleic acid | developmental process: embryonic axis; fin morphogenesis | nucleus |
| **mir_9** | zgc:85707 | Sprouty-related protein 1 with EVH-1 domain | target 5' A C G C 3'  AGCUG UA AAACAAUCA  UCGAC AU UUUGUUAGU  miRNA 3' UGUG U G 5' | -24,4 | binding | Developmental process: multicellular organismal process | membrane |
| **mir_9** | nr2f1l | Nuclear receptor protein | target 5' A C A 3'  ACACG UGAU UAAACAAUCA  UGUGU ACUA GUUUGUUAGU  miRNA 3' CG U 5' | -23,7 | binding: DNA | Cellular process: metabolic | nucleus |
| **mir_10** | sec23b | SEC23B | target 5' A UUU G G 3'  GUG GAAU AUACCCCU  CGC CUUA UAUGGGGA  miRNA 3' U G 5' | -25,3 | binding: protein | developmental process: neurocranium morphogenesis; cartilage development | cytoplasm |
| **mir_10** | ptpra | protein tyrosine phosphatase, receptor type | target 5' A A CUC U 3'  GC GG AAUCAUACCCCU  CG UC UUAGUAUGGGGA  miRNA 3' C 5' | -24,6 | catalytic activity | cellular process: metabolic | unknown |
| **mir_10** | myst3 | MYST histone acetyltransferase (monocytic leukemia) 3 | target 5' A U A A 3'  GC GAGAG CGUACCCC  CG CUCUU GUAUGGGG  miRNA 3' A A 5' | -26,7 | binding: DNA | developmental process: embryonic patterning; cartilage and skeletal development | nucleus |
| **mir_10** | parva | parvin, alpha | target 5' U U UU A 3'  GC G AAUUAUACCCCU  CG C UUAGUAUGGGGA  miRNA 3' UC 5' | -23,8 | unknown | unknown | unknown |
| **mir_10** | exoc6 | exocyst complex component 6 | target 5' A CA G 3'  GUGAGG AUACCUU  CGCUCU UAUGGGG  miRNA 3' UAG A 5' | -19,2 | unknown | cellular process: exocytosis | cytoplasm |
| **mir_10** | pgm1 | phosphoglucomutase 1 | target 5' C UU A 3'  AG UCAUACCCC  UC AGUAUGGGG  miRNA 3' CGC UU A 5' | -23,1 | catalytic activity | Cellular process: metabolic | unknown |
| **mir_13** | zar1 | Zygote arrest 1 (Oocyte-specific maternal effect factor) | target 5' U UAC U 3'  UG CAG UUGUAGGUGUGA  AC GUC AACAUCCACACU  miRNA 3' G CCU 5' | -26,9 | binding: ion | unknown | Unknown |
| **mir_13** | rbpms2 | Cytoplasmic polyadenylation element-binding protein 1 | target 5' A U G 3'  G CAGG GUUGUGGGUGUGA  C GUCC UAACAUCCACACU  miRNA 3' A G C 5' | -32,7 | binding: nucleotide | unknown | unknown |
| **mir_14** | tnrc4 | trinucleotide repeat containing 4 | target 5' G GAG U 3'  UCAGUUAC UCACUUU  AGUCAGUG AGUGAAA  miRNA 3' CAG GAA 5' | -23,4 | binding: nucleotide | unknown | unknown |
| **mir_14** | timp2 | tissue inhibitor of metalloproteinase 2 | target 5' A CUA A 3'  CUUA ACCUUUCACUU  GAGU UGGAAAGUGAA  miRNA 3' CA CAG A 5' | -23,8 | unknown | unknown | unknown |
| **mir_14** | per4 | period homolog 4 | target 5' G CU G C 3'  CUC GUUACC UUCACUUU  GAG CAGUGG AAGUGAAA  miRNA 3' CA U A 5' | -24,6 | molecular transducer activity | cellular process: signal transduction | unknown |
| **mir_14** | rab6b | RAB6B, member RAS oncogene family | target 5' U 3'  UCUC UUAUCUUUCACUUU  AGAG AGUGGAAAGUGAAA  miRNA 3' C UC 5' | -24,5 | binding: nucleotide | cellular process: signal transduction | membrane |
| **mir_14** | crsp8 | cofactor required for Sp1 transcriptional activation, subunit 8 | target 5' U CA A G 3'  UCUC UCAU UUUUUACUU  AGAG AGUG GAAAGUGAA  miRNA 3' C UC A 5' | -19,1 | unknown | developmental process: eye | unknown |
| **mir_14** | tbx6 | T-box transcription factor TBX6 | target 5' G G GG G 3'  CUCAG CAC UUCACUU  GAGUC GUG AAGUGAA  miRNA 3' CA A GA A 5' | -25,1 | binding: nucleotide | biological regulation: transcription | nucleus |
| **mir_14** | melk | maternal embryonic leucine zipper kinase | target 5' G U G A 3'  CUCA UCAUUU UCACUUU  GAGU AGUGGA AGUGAAA  miRNA 3' CA C A 5' | -22,8 | Binding: nucleotide | biological regulation (heart contraction) | unknown |
| **mir_15** | zgc:85956 | transcription factor 12 | target 5' C UC C 3'  CUCAGC ACAUA GCUGUCUG  GGGUUG UGUAU UGAUGGAU  miRNA 3' U UU 5' | -22,1 | binding: nucleotide | biological regulation: transcription | nucleus |
| **mir_16** | cyp24a1l | cytochrome P450, family 24, subfamily A, polypeptide 1, like | target 5' C A 3'  GAGGCCUU UAUAAUGCUGCU  UUUCGGGA AUGUUACGACGA  miRNA 3' C 5' | -32,4 | unknown | unknown | unknown |
| **mir_16** | gfm2 | G elongation factor, mitochondrial 2 | target 5' U G U U 3'  GCUCUGUA A UGCUGCU  CGGGACAU U ACGACGA  miRNA 3' UUU G U 5' | -30,1 | binding: nucleotide | unknown | unknown |
| **mir_16** | vox | ventral homeobox | target 5' C UA U 3'  AA UCUGUACAGUGCUGCU  UU GGACAUGUUACGACGA  miRNA 3' U CG 5' | -32,2 | Binding: DNA | developmental process: dorsal/ventral pattern formation | nucleus |
| **mir_20** | elf2 | E74-like factor 2 (ets domain transcription factor) | target 5' G C C 3'  CAUUCU AC CAUCCCAAUCU  GUGGGA UG GUGGGGUUAGA  miRNA 3' U A 5' | -28,1 | binding: nucleotide | biological regulation: transcription | nucleus |
| **mir_20** | ubin | ataxin-1 ubiquitin-like interacting protein | target 5' C G A 3'  UCCUAAUUCAU CCUGAUC  GGGAUUGAGUG GGGUUAG  miRNA 3' GU A 5' | -27,2 | catalytic activity | cellular process: protein modification | unknown |
| **mir_21** | rnf11 | ring finger protein 11 | target 5' A C A G A C 3'  UGACUC G AUU A AUGUAGCU  ACUGGG C UAA U UACAUCGA  miRNA 3' U A G C 5' | -24,1 | Binding: ion; protein | establishment of localization: zinc transport | unknown |
| **mir_22** | col9a2 | type IX collagen alpha 2 precursor | target 5' U CCUCA A 3'  GUCCCA CAGUACAGAGAG  CAGGGU GUCGUGUCUCUC  miRNA 3' UUACC C 5' | -34,6 | unknown | establishment of localization: phosphate transport | cytoplasm |
| **mir_23** | msto1 | misato homolog 1 | target 5' A G C AG G 3'  G CAUG UGGG GAAUUAU  U GUAU ACUC CUUAGUA  miRNA 3' G G AG 5' | -19,3 | unknown | unknown | unknown |
| **mir_23** | prkar1a | protein kinase, cAMP-dependent, regulatory, type I, alpha | target 5' C A UU G A 3'  CAUCA AUG U GGAUUA  GUGGU UAC A CUUAGU  miRNA 3' A UC G A 5' | -17,2 | enzyme regulator activity | cellular process: protein amino acid phosphorylation | intracellular part |
| **mir_23** | skib | nuclear oncoprotein skib | target 5' U A AC G 3'  UACCA AUGA UGAAUCAU  GUGGU UACU GCUUAGUA  miRNA 3' A CA 5' | -21,4 | catalytic activity | Cellular process: metabolic | nucleus |
| **mir_25** | gtpbp1l | GTP binding protein 1, like | **position 772**  target 5' U CUG C U 3'  GAC UA GGGCCACUU  CUG AU UCCGGUGAA  miRNA 3' AU AAA C 5' | -23,3 | binding: nucleotide; GTP | unknown | unkown |
| **mir_25** | trpa1a | transient receptor potential cation channel, subfamily A, member 1a | target 5' C C AAU G 3'  AC UUUA GCCACU  UG AAAU CGGUGA  miRNA 3' AUC A CUC A 5' | -16,0 | Transporter activity | Developmental process: multicellular organismal process | membrane |
| **mir_25** | tbx1 | T-box transcription factor TBX1 | target 5' C AAUGAA G 3'  AGAC AGGCCACU  UCUG UCCGGUGA  miRNA 3' A AAAAUC A 5' | -23,8 | Binding: DNA | cellular process: transcription | nucleus |
| **mir_25** | eif4b | Eif4b protein | target 5' A UAAA C 3'  GGACUUUU GCCACU  UCUGAAAA CGGUGA  miRNA 3' A UCUC A 5' | -23,7 | Binding: nucleotide | unkown | unkown |
| **mir_25** | nmt1 | N-myristoyltransferase 1 | target 5' C UG A G 3'  AG UUUUGGA GCCACU  UC AAAAUCU CGGUGA  miRNA 3' A UG C A 5' | -28,2 | catalytic activity | cellular process: metabolic | unknown |

The antisense miRNA sequence for the novel miRNAs were blasted against the ZF 3’UTRs. Sequences with perfect seed match between nucleotides 2 and 7, and no more than 6 mismatches in the remaining sequence, were retained for further analysis. Targets obtained by this method were considered whenever RNAhybrid confirmed them thermodynamically. In the cases where RNAhybrid was not capable of retrieving the same target obtained by the first approach, it was discarded.
